# Evaluating the value of tumor length times width in colorectal adenocarcinoma with different tumor locations

**DOI:** 10.1097/MD.0000000000029845

**Published:** 2022-06-30

**Authors:** Encheng Zhou, Jianhui Chen, Shuwang Peng, Jingfeng Chen, Ting Fei, Xiaojun Wang, Changlei Qi, Qing Huang

**Affiliations:** a Department of Gastrointestinal Surgery of The Affiliated Hospital of Medical School, Ningbo University, Ningbo, Zhejiang, China; b Department of Gastrointestinal Surgery, Taizhou Hospital, Wenzhou Medical University, Linhai, Zhejiang, China; c Department of General Surgery of the First Hospital of Hunan University of Chinese Medicine, Changsha, Hunan, China; d Anus and intestine surgery department of Central Hospital of Lishui, Lishui, Zhejiang, China; e Emergency Department of The Affiliated Hospital of Medical School, Ningbo University, Ningbo, Zhejiang, China.

**Keywords:** colorectal cancer, primary tumor location, prognosis, tumor size, tumor length times width (TLTW), tumor width

## Abstract

The T classification, which reflects the vertical growth pattern of the tumor, is one of the most important prognostic factors in colorectal cancer. We aimed to investigate the prognostic value of tumor length and width in patients with colorectal cancer (CRC).

A total of 259 patients with stage I–III CRC who underwent curative resection were reevaluated according to tumor location. One-way ANOVA analysis was conducted to investigate the relationship between the tumor length times width (TLTW) and clinical parameters. Univariate and multivariate analyses were conducted to analyze the potential prognostic factors affecting overall survival (OS) of patients with stage I–III CRC. In the entire cohort, the TLTW was analyzed as a continuous variable.

The results suggested that TLTW (*P* = .003) and tumor location (*P* = .04) could be independent prognostic factors for patients with CRC. In addition, TLTW had an intimate relationship with tumor location (*P* < 0.001) and differentiation (*P* = .003). The mean TLTW of the right colon was significantly larger than mean TLTW of the left colon and rectal cancers. However, the mean TLTW of the left colon cancer was similar to that of the rectal cancer TLTW (*P* > 0.05, not shown). Subgroup analysis of TLTW according to tumor location suggested that TLTW was an independent prognostic factor for patients with right colon cancer (RCC) (*P* = .007) rather than left colon cancer (LCC) (*P* = .49) or rectal cancer (*P* = .16). Kaplan-Meier (K-M) analysis based on tumor location suggested that the survival rate of RCC patients had a distinctly higher trend rate than LCC patients and RECC patients in the long-term rather than in the short-term.

TLTW is closely associated with tumor location in CRC. In addition, TLTW may be an independent prognostic factor for patients with RCC.

## 1. Introduction

The incidence of colorectal cancer (CRC) is increasing, and it is the fourth most commonly diagnosed malignancy and the fifth most common cause of mortality among tumor sufferers in China.^[1]^ The prognosis of patients with CRC has improved significantly over the past few decades owing to the application of advanced surgical techniques and superior postoperative chemotherapy regimens. The tumor-node-metastasis (TNM) staging system, the gold standard for various types of cancer, is currently the most significant factor for evaluating the prognosis of CRC patients. According to TNM stage, the T subcategory in CRC refers to vertical tumor penetration within or beyond the bowel wall rather than the maximum horizontal tumor diameter. The T stage of many solid tumors, such as renal,^[2]^ breast,^[3]^ and lung cancers^[4]^ is confirmed by tumor size, defined as the longest horizontal tumor diameter, namely tumor length. However, the T classification of CRC refers to the bowel wall layers that are vertically infiltrated by the tumor rather than the tumor size. Recently, the prognostic role of tumor length, one of the indicators reflecting the tumor extent, was again appraised in a series of studies in CRC.^[4–8]^ However, these results indicated that the prognostic impact of tumor size remains controversial and needs further investigation. This phenomenon may be due to the fact that the tumor size of CRC alone is not able to reflect the actual degree of tumor growth, thus making it difficult to assess the prognosis of CRC patients. In addition, the prognostic value of the widest tumor width perpendicular to the tumor size was overlooked. Consequently, on the basis of existing literature data, we took advantage of tumor length times width (TLTW), aiming to reflect the degree of tumor growth more accurately, thus yielding a better prognosis evaluation for CRC patients.

Accumulating evidence has verified that CRC characteristics such as epidemiology, pathological characteristics, and clinical outcomes differ according to primary tumor locations.^[9–12]^ The type of surgical operation of colorectal cancer is also based on the location of the tumor, namely right hemicolectomy, extended right hemicolectomy, left hemicolectomy, and anterior resection. The surgical approach according to the tumor location has been shown to have a great impact on the prognosis of CRC. In addition, further evidence indicates that embryological origins, histology, anatomy, genetics, and immunology of right colon cancer (RCC) differ from those of left colon cancer (LCC) and rectal cancer.^[11,13]^ At present, the expression of certain genes in colorectal cancer is closely related to the prognosis of the tumor, such as *K-RAS*, N*- RAS*, *B-RAF*, *MMR*, and *EGFR*. Furthermore, many studies have been conducted to verify the prognostic value of tumor size, whereas few previous studies have considered the primary tumor location, and to date, no consensus has been reached.^[5–8,14–16]^ Cai et al demonstrated that the tumor size of the colon is larger than that of rectal cancer.^[10]^ According to Kornprat et al, colon tumor size was significantly correlated with tumor location, and the optimal cut-off values of colon cancer decreased from the RCC to LCC, and ultimately to the rectum.^[17]^ Those results suggested the tumor extent may also vary according to different primary tumor locations. Therefore, the impact of primary tumor location should not be neglected when estimating the prognostic value of clinical parameters, including factors reflecting tumor dimension, such as tumor length and width.

In the present study, we aim to investigate the relationship between TLTW and clinical parameters of CRC patients. In addition, we further explore the prognostic role of TLTW for CRC patients in accordance with primary tumor locations.

## 2. Methods

### 2.1. Setting the value of TLTW

**Figure 1.**
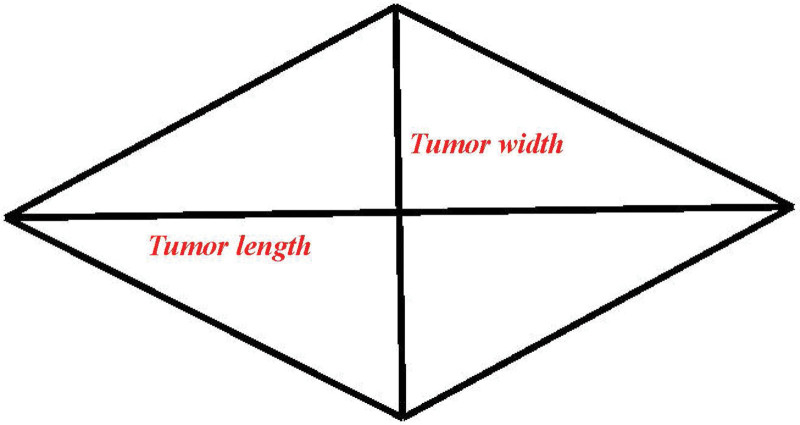
Sketch map of tumor length and tumor width in our study.

**Figure 2.**
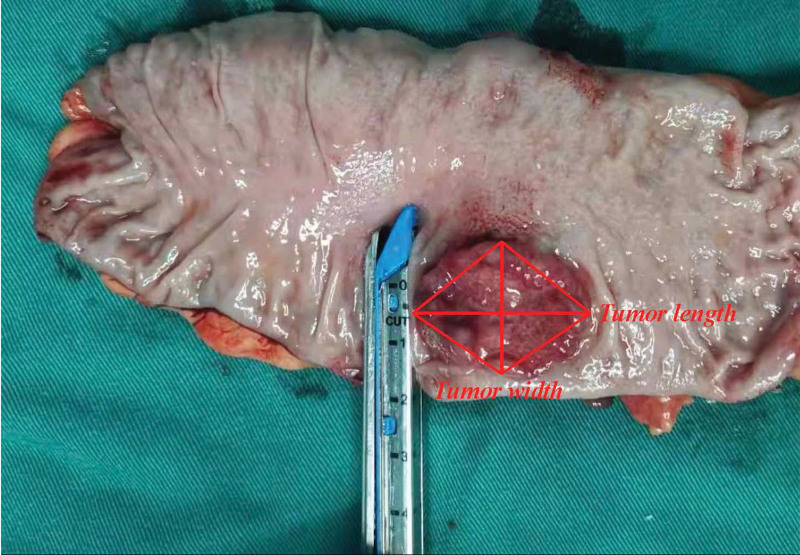
Sketch map of setting tumor length and tumor width in tumor specimens.

In our study, tumor length was defined as the longest horizontal tumor diameter, that is, tumor size. The longest horizontal tumor diameter perpendicular to the tumor size was defined as tumor width. In this study, we regarded the tumor shape as rhombus, as shown in the sketch map in Figure 1. Moreover, we obtained a tumor specimen and set the tumor length and width for presentation (Fig. 2). We then multiplied the tumor length and width to obtain the TLTW value.

### 2.2. Patients

This study was approved by the Ethics Committee of the Affiliated Hospital of the Medical School of Ningbo University. Written informed consent was obtained from all the patients in this study. A total of 259 patients with CRC who underwent initial resection at the Hospital Affiliated to Ningbo University School of Medical from 2005 to 2017 were recruited. Patients with stage IV disease were excluded from this study. Tumor location including the cecum/appendix, ascending colon, hepatic flexure, and proximal transverse colon (two-thirds of the proximal transverse colon), was defined as RCC. Tumor location including the distal transverse colon (distal one-third of the transverse colon), splenic flexure, descending colon, and sigmoid colon, was defined as LCC. Rectal cancer refers to rectosigmoid colon cancer to the dentate line.^[17]^ The patients’ ages ranged from 26 to 90 years (median age, 65 years). All selected colon cancer tissues met the following inclusion criteria: (1) the patient underwent curative resection; (2) the patient had a regular follow-up; (3) patients with pathologically confirmed colorectal cancer; and (4) patients with stage I, II, or III CRC. Exclusion criteria were (1) history of previous malignant disease or a second primary tumor, (2) familial adenomatous polyposis, (3) preoperative chemotherapy and/or radiation, and (4) synchronous distant metastases at diagnosis.

### 2.3. Data collection

The following clinical variables were collected in this study: age at diagnosis, sex, differentiation, primary tumor location, cancer embolus, breakthrough serosa, lymph node metastasis, TLTW, depth of invasion, lymph metastasis, American Joint Committee on Cancer (AJCC) stage (TNM), and survival time. The tumor length was defined as the maximum horizontal tumor diameter. Similarly, tumor width was defined as the widest horizontal tumor diameter perpendicular to the tumor length. Tumor length and width were collected from the pathology reports of the resected CRC specimens. The length and width were then multiplied for the study. In addition, the products of the length multiplied by width were regarded as continuous variables in both univariate and multivariate analyses, and other parameters were analyzed as categorical variables. The primary tumor location was defined as RCC, LCC, or RECC. The stage of all patients was defined according to the seventh edition of the AJCC Cancer Staging Manual.

### 2.4. Follow-up

In the first year, the patients were examined at the hospital every 3 months. For the second year, patients were examined every 6 months, and annually thereafter. Laboratory checks, Computed Tomography (CT) scans, and other examinations were performed according to the CRC treatment guidelines. The primary endpoint of this study was overall survival (OS), defined as the period from 1 month later after surgery to death from any cause. The secondary endpoint was follow-up.

### 2.5. Statistical analysis

SPSS software (version 18.0, IBM) was used to analyze the research data. One-way analysis of variance (ANOVA) tests were used to analyze the association between TLTW and clinical variables. The Cox proportional hazards model was used in the multivariate analysis of prognostic factors. The Kaplan-Meier method was used to investigate the overall survival rate of patients with colorectal cancer according to tumor location. Statistical significance was set at *P* < 0.05.

## 3. Results

### 3.1. Patient characteristics

We recruited 259 patients with stage I–III who underwent initial curative resection at Taizhou Hospital affiliated with Wenzhou Medical University. The clinical parameters of this study are summarized in Table 1. The age range was 26 to 90 years (mean age, 65 years). Of these, 57.1% (148/259) were male and 42.9% (111/259) were female. The proportions of RCC, LCC, and RECC were 37.5% (97/259), 25.5% (66/259), and 37.1% (96/259), respectively. The maximum and minimum values of TLTW were 0.15 cm^2^ and 121.00 cm^2^, and the median was 20.00 cm^2^. As shown in Table 2, the follow-up duration ranged from 2 to 96 months, with a median of 41 months.

**Table 1:** Correlation between TLTW and clinicopathologic parameters in patients with colorectal cancer (n = 259).

Clinicopathologic parameters	Patients (n)	Percent	Mean (cm^2^)	F	*P*
Age					
≤median (65 years)	126	48.6%	25.35	0.239	0.63
>median (65 years)	133	51.4%	24.28		
Gender				0.239	0.63
Male	148	57.1%	24.72		
Female	111	42.9%	24.92		
Differentiation				4.676	0.003
Well	4	1.5%	10.47		
Middle	187	72.2%	22.87		
Poor	37	14.3%	30.39		
Mucinous adenocarcinoma	31	12%	31.65		
Cancer embolus				1.271	0.26
No	189	73%	25.55		
Yes	70	27%	22.79		
Breakthrough serosa				2.580	0.11
No	113	43.6%	22.82		
Yes	146	56.4%	26.33		
Lymph node metastasis				1.671	0.20
No	141	54.4%	26.09		
Yes	118	45.6%	23.27		
Depth of invasion				1.595	0.19
T1	2	0.8%	11.50		
T2	18	6.9%	18.84		
T3	87	33.6%	23.67		
T4	152	58.7%	26.33		
Lymph metastasis				1.103	0.33
N0	136	52.7%	26.28		
N1	72	27.9%	22.54		
N2	50	19.4%	24.28		
TNM				2.561	0.08
I	17	6.6%	18.61		
II	121	46.7%	27.12		
III	121	46.7%	23.36		
Patients’ survival				2.312	0.13
No	60	23.2%	27.81		
Yes	199	76.8%	23.90		
Tumor location				16.028	<.001
Right	97	37.5%	32.34		
Left	66	25.5%	20.23		
Rectum	96	37.1%	20.33		

**Table 2:** Univariate and Multivariate Cox regression analysis of OS in patients with CRC (n = 259).

Clinicopathologic parameters	Patients (n)	8-years survival patients (n)		Univariate Analysis P	Multivariate analysis P
		No	Yes		
Age				0.235	0.57
≤median (65 years)	126	29	97		
>median (65 years)	133	31	102		
Gender				0.162	0.11
Male	148	39	109		
Female	111	21	90		
Differentiation				0.151	0.03
Well	4	0	4		
Middle	187	44	143		
Poor	37	11	26		
Mucinous adenocarcinoma	31	5	26		
Cancer embolus				0.003	0.01
No	189	154	35		
Yes	70	45	25		
Breakthrough serosa				0.002	0.11
No	113	97	16		
Yes	146	102	44		
Lymphnode metastasis				0.002	0.66
No	141	119	22		
Yes	118	80	38		
T stage				0.008	0.84
T1	2	2	0		
T2	18	16	2		
T3	87	73	14		
T4	152	108	44		
N stage				0.000	0.006
N0	136	116	20		
N1	72	54	18		
N2	50	29	21		
TNM				0.001	0.77
I	17	16	1		
II	121	101	20		
III	121	82	39		
TLTW	259	199	60	0.130	0.003
Tumor location				0.000	0.04
Right	97	79	18		
Left	66	46	20		
Rectum	96	74	22		

**Table 3:** Univariate and multivariate Cox regression analysis of OS in CRC patients according to tumor location (n = 259).

Clinicopathologic parameters	Right-side (n = 97)			Left-side (n = 66)			Rectal cancer (n = 96)		
	Patients(n)	Mean (cm^2^)	*P*	Patients(n)	Mean (cm^2^)	P	Patients(n)	Mean (cm^2^)	P
Age			0.395			0.258			0.814
≤median(65y)	55	30.62		34	21.94		37	20.66	
>median (65y)	42	34.59		32	18.41		59	20.13	
Gender									.113
Male	48	31.32	0.665	42	21.29	0.366	58	21.73	
Female	49	33.33		24			38	18.20	
Differentiation			0.298			0.020			0.053
Well	2	10.50		1	12.15		1	27.38	
Middle	60	22.14		53	18.37		74	19.76	
Poor	19	34.27		6	24.12		12	27.37	
Mucinous adenocarcinoma	16	39.01		6	34.12		9	16.92	
Cancer embolus			0.494			0.709			0.860
No	75	33.20		47	20.60		67	20.46	
Yes	22	29.41		19	19.31		29	20.04	
Breakthrough serosa			0.983			0.482			0.082
No	28	32.42		31	21.39		54	18.67	
Yes	69	32.31		35	19.20		42	22.47	
Lymph node metastasis			0.853			0.455			0.636
No	61	32.67		37	21.26		43	20.91	
Yes	36	31.78		29	18.91		53	19.86	
Depth of invasion			0.557			0.333			0.405
T1	1	14.00		1	9.00		–	–	
T2	1	6.00		3	16.50		14	20.27	
T3	24	31.82		24	23.66		39	18.68	
T4	71	33.34		38	18.66		43	21.86	
Lymph metastasis			0.996			0.615			.586
N0	60	32.40		35	21.68		41	21.256	
N1	20	32.55		22	18.72		30	18.678	
N2	17	31.89		9	18.31		25	20.820	
TNM			0.375			0.606			0.735
I	2	10.00		3	17.00		12	20.46	
II	59	32.84		33	21.75		29	21.58	
III	36	32.75		30	18.88		55	19.65	
Patients’ survival			0.031			0.474			0.060
No	18	42.67		20	20.97		22	19.22	
Yes	79	29.42		46	18.54		74	24.08	

### 3.2. Relationship between mean TLTW and clinicopathologic parameters

The correlation between TLTWs and various clinicopathological parameters, as shown in Table 1, was conducted among 259 CRC patients. The results revealed that the mean TLTW significantly correlated with tumor location (*P* < 0.001) and differentiation (*P* = .003) in CRC. However, the TLTWs were not significantly correlated with age (*P* = .63), sex (*P* = .63), cancer embolus (*P* = .26), depth of invasion (*P* = .11), breakthrough serosa (*P* = .11), lymph node metastasis (*P* = .20), lymph metastasis (*P* = .33), TNM stage (*P* = .08), and survival time (*P* = .13). Notably, the results indicated that TLTWs in RCC were significantly larger than those in LCC and rectal cancer. However, the mean TLTW in left-sided colon cancer was not significantly different from that of rectal cancer. In addition, the results demonstrated that the TLTWs of CRC were significantly associated with tumor differentiation (*P* = .003). The mean TLTW of well differentiation was smaller than that of moderate differentiation, and the mean TLTW of moderate differentiation was smaller than that of poor differentiation. In addition, there were no significant differences between poor differentiation and mucinous adenocarcinoma in the TLTWs.

### 3.3. Univariate and multivariate analysis of clinical parameters

As shown in Table 2, univariate analyses of OS indicated that cancer embolus (*P* = .003), breakthrough serosa (*P* = .002), lymph node metastasis (*P* = .002), tumor location (*P* < 0.001), T stage (*P* = .008), N stage (*P* = .000), and TNM stage (*P* = .001) were poor prognostic factors for CRC patients. Multivariate analysis of OS indicated that tumor location (*P* = .04), differentiation (*P* = .03), cancer embolus (*P* = .01), N stage (*P* = .006), and TLTW (*P* = .003) were independent risk factors for OS of patients with CRC.

### 3.4. Subgroup analysis and the prognostic value of TLTW according to tumor location

To evaluate the prognostic role of TLTW in different colorectal cancer sites, subgroup analysis according to tumor location was conducted. As shown in Table 3, the results suggested that lymph node metastasis (*P* = .03), N stage (*P* = .04), and TLTW (*P* = .007) were significantly associated with the survival rate of RCC patients. Overall, the results suggest that TLTW could be an independent prognostic factor for patients with RCC. The results suggested that TLTW had no significant association with overall survival rate in patients with LCC and RECC. When we combined patients with LCC or RECC into 1 subgroup, the results showed that TLTW had no significant association with survival time for patients with left-sided colorectal cancer (results not shown).

### 3.5. Overall survival according to primary tumor location

**Figure 3.**
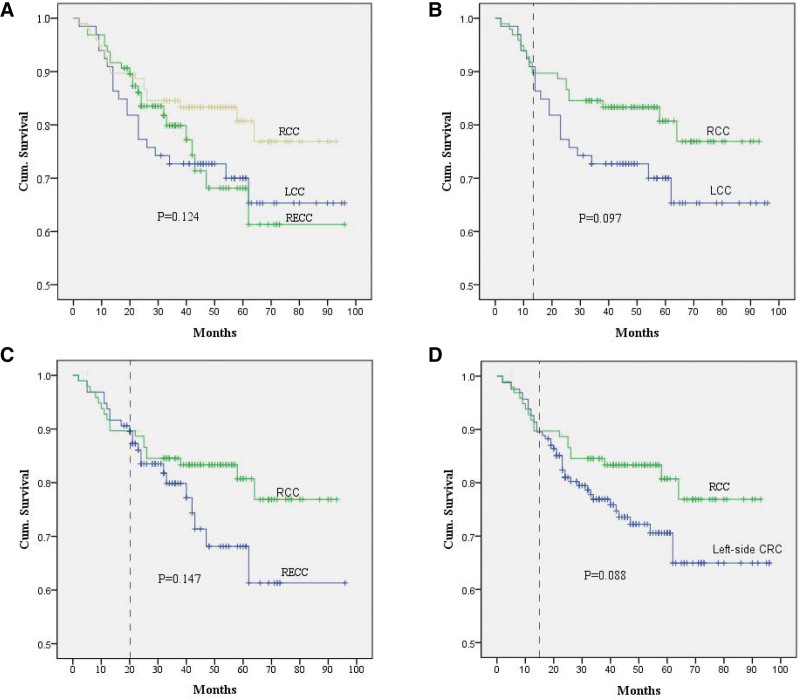
The Kaplan-Meier survival analysis comparative curves in accordance with tumor location. RCC, LCC and RECC (A); RCC and LCC (B); RCC and RECC (C); RCC and Left-side CRC (including LCC and RECC) (D).

All 259 patients were followed up approximately 1 month after surgery. The postfollow-up period lasted 100 months, and the sheathed stage ranged from 2 to 96 months (median 41 months). During the course of the study, 60 (23.17%) patients with CRC died. Among these deaths, 18 (18.56%,18/97), 20 (30.30%, 20/66), and 22 (22.92%, 22/96) deaths occurred in patients with RCC, LCC, and RECC, respectively. Among these deaths, the 8-year survival rate of patients with colorectal cancer, analyzed in accordance with tumor location, did not show a significant difference (*P* = .12, log-rank test) (Fig. 3A). The long-term survival rate of patients with RCC was distinctly higher than patients with LCC (*P* = .10, log-rank test) (Fig. 3B) and RECC (*P* = .15, log-rank test) (Fig. 3C). However, the survival rate of RCC patients was not different from that of LCC and RECC patients in the short-term (Fig. 3). When integrating the LCC and RECC together, the results suggested that the survival rate of RCC patients has a distinctly higher trend than left-sided colorectal cancer patients in the long-term (*P* = .09, Fig. 3D). The comparison of survival rates in RCC, LCC, and RECC requires further study.

## 4. Discussion

Tumor size plays an important role in the prognostic evaluation system of many solid cancers. Tumor length and width are simple horizontal growth index metrics that are standardized in different hospital systems and are usually reported in routine pathological examinations. However, although many efforts have been made to illuminate the prognostic value of the largest horizontal tumor extent, the results were controversial.^[5–8,14–16]^ Numerous investigators have confirmed that tumor size is a negative risk factor for patients with CRC.^[8,17–19]^ However, many other studies have suggested that tumor size could not be an independent prognostic effect in multivariate analysis.^[20–23]^ In addition, many subgroup studies identifying the prognostic role of tumor size based on various clinicopathological parameters have also been conducted, such as TNM stage,^[5,24]^ tumor necrosis,^[25]^ and tumor macroscopic growth pattern.^[26]^ In addition, a series of methods, including receiver operating characteristic curve (ROC) statistics^[7,17]^ and X-tile programs,^[16]^ have been implemented to determine the significant cut-off points of tumor size, the results of which suggest that the cut-off points for tumor size are not widely applicable. Consequently, in our retrospective analysis, we first combined the tumor length and width by multiplying them, and the results showed that TLTW was significantly associated with the prognosis of CRC patients. The conclusion of this study is inconsistent with those of some previous studies, which may be a reason why tumor size, as opposed to TLTW, cannot accurately reflect the degree of tumor growth.

Evidence suggests that RCC and LCC are derived from different parts of the gut.^[27]^ Although the rectum is also derived from the same part of the gut as the left-sided colon, RECC has occasionally been investigated separately. Consequently, CRC was classified into 3 subgroups based on their primary tumor location, namely RCC, LCC and RECC.^[28]^ Moreover, increasing evidence has shown that the epidemiology, pathological features, and clinical outcomes of CRC are different in accordance with primary tumor locations.^[9–12]^ Modest et al inferred that stage III RCC patients had significantly shorter progression-free survival and OS than stage III LCC patients.^[29]^ Recently, in a meta-analysis of 66 clinical studies with 1.4 million patients, tumor location was shown to have a significant association with prognosis of patients with CRC.^[30]^ Based on accumulated findings, the issue of whether to consider CRC as 3 separate tumor entities according to anatomical site has been discussed. Interestingly, Takahashi et al demonstrated the most striking result that the optimal cut-off value of tumor size with respect to outcome decreases from the right colon to the left colon, with the smallest cut-off value for rectal cancer.^[31]^ Similarly, Moda et al showed that the mean tumor size on the right side is significantly larger than that in left-sided cancers (6.1 vs 4.8 cm).^[32]^ In a study analyzing the distribution characteristics of tumor location and tumor size among 3369 Chinese colorectal cancer patients during colonoscopy, the results also showed that colon cancer was significantly larger than rectal cancer.^[10]^ Recently, Lim et al also verified that RCC exhibited a greater average tumor size than LCC.^[33]^ Remarkably, our current study similarly revealed that TLTW had a significant correlation with tumor anatomical location (*P* = .000). Our results showed that the mean TLTW in RCC was significantly larger than mean TLTW in LCC and RECC. However, the mean TLTW in LCC was not significantly different from the mean TLTW in RECC. Based on the above evidence, we investigated the prognostic value of TLTW for patients with CRC based on the tumor site. This is a reasonable way to eliminate the effect of tumor location on TLTW when analyzing the prognostic role of TLTW in patients with CRC. In our current study, the results indicated that TLTW was an independent prognostic factor for RCC patients but not for LCC and RECC patients. Moreover, our results also suggested that tumor location could be an independent prognostic factor for CRC patients (*P* = .000). In addition, our K-M survival analysis suggested that the prognosis of RCC patients was better than that of LCC and RECC patients in the long-term when compared with the short-term. Our results are correlate somewhat with their investigation.^[34,35]^ Consequently, it is reasonable to consider RCC, LCC, and RECC as 3 separate tumor entities and consider the prognostic role of clinical factors based on tumor location, especially for factors reflecting tumor growth extent, such as tumor size, TLTW, and tumor volume.

To our knowledge, this is the first study to demonstrate the prognostic value of TLTW in CRC with different primary tumor locations. However, the present study has several limitations. First, our investigation was a single-center retrospective study, and the number of recruited CRC patients was relatively small (n = 259). Second, adjuvant chemotherapy, laboratory examinations, and other vital factors were not included in this study. Third, the optimal cut-off value of TLTW in accordance with the primary tumor location was not determined. Nevertheless, the current study has a significant advantage in that we identified a novel prognostic factor for patients with RCC. In addition, our study provides a new perspective for investigating the prognostic role of tumor size. Further multicenter prospective investigations on TLTW should be conducted, and additional baseline parameters should be considered.

In conclusion, TLTW, which reflects the extent of tumor growth, was closely associated with tumor location. TLTW was an independent factor for OS in patients with stage I–III RCC but not in patients with LCC and RECC. In addition, we should consider the prognostic role of clinical factors based on tumor location, especially for factors reflecting the extent of tumor growth. In the future, we will continue to study the role of TLTW in CRC patients.

### Author contributions

Encheng Zhou, Jianhui Chen, and Shuwang Peng collected the clinical samples. Jianhui Chen and Ting Fei analysed the data. Qing Huang, Changlei Qi, and Encheng Zhou conceived and supervised the project, analysed the data and drafed the manuscript. Encheng Zhou, Jingfeng Chen, and Xiaojun Wang analysed the data and helped write the manuscript. All authors have approved the final article.
